# Real-time PCR analysis of fungal organisms and bacterial species at peri-implantitis sites

**DOI:** 10.1186/s40729-015-0010-6

**Published:** 2015-04-21

**Authors:** Frank Schwarz, Kathrin Becker, Sebastian Rahn, Andrea Hegewald, Klaus Pfeffer, Birgit Henrich

**Affiliations:** 1 Department of Oral Surgery, Westdeutsche Kieferklinik, Heinrich Heine University, Moorenstraße 5, D-40225 Düsseldorf, Germany; 2 Department of Orthodontics, Westdeutsche Kieferklinik, Heinrich Heine University, Moorenstraße 5, D-40225 Düsseldorf, Germany; 3 Institute of Medical Microbiology and Hospital Hygiene, Heinrich Heine University, Universitätsstraße 1, D-40225 Düsseldorf, Germany

**Keywords:** Peri-implantitis, Diagnosis, Microbiology, Fungal organisms

## Abstract

**Background:**

The potential role of fungal organisms and their co-aggregation with either periodontopathogens or opportunistic pathogens at peri-implantitis sites is unknown. The aim of the present study was to qualitatively/quantitatively analyze and correlate fungal organisms and bacterial species at peri-implantitis sites.

**Methods:**

In a total of 29 patients, submucosal/subgingival plaque samples were collected at peri-implantitis and healthy implant sites as well as teeth with a history of periodontitis (controls). A real-time PCR assay was established for the qualification of fungal organisms and a TaqMan assay for the quantification of *Porphyromonas gingivalis*, *Parvimonas micra*, *Tannerella forsythia*, *Mycoplasma salivarium*, *Veillonella parvula*, and *Staphylococcus aureus*.

**Results:**

Fungal organisms were more frequently identified at peri-implantitis (31.6%) (i.e., *Candida albicans*, *Candida boidinii*, *Penicillium* spp*.*, *Rhodotorula laryngis*, *Paelicomyces* spp*.*, *Saccharomycetes*, *Cladosporium cladosporioides*) and healthy implant sites (40% - *Candida dubliniensis*, *C. cladosporioides*) than at selected teeth (20% - *C. albicans*, *Fusarium solani*). At implant sites, fungal organisms were significantly correlated with *P. micra* and *T. forsythia*.

**Conclusions:**

*Candida* spp*.* and other fungal organisms were frequently identified at peri-implantitis as well as healthy implant sites and co-colonized with *P. micra* and *T. forsythia*.

## Background

There is considerable evidence supporting the view that peri-implant diseases are infectious in nature and mainly linked to an uncontrolled accumulation of bacterial plaque biofilms [1]. Basically, diseased implant sites are dominated by gram-negative anaerobic bacteria and therefore feature microbiological characteristics similar to those noted for chronic periodontal infections [2]. Even though the history of periodontitis is a documented risk indicator for peri-implant diseases [3,4], the diversity of microbiota at diseased tooth sites was reported to be higher than that noted at diseased implant sites [5]. Common periodontopathogenic bacteria could be isolated at both healthy and diseased implant sites [6], and the microbiological analysis of 40 species did not markedly differ by the clinical implant status (i.e., healthy, mucositis, peri-implantitis) [7]. However, a most recent analysis of 78 species has pointed to higher counts of 19 bacterial species at peri-implantitis - when compared with healthy implant sites, mainly including *Porphyromonas gingivalis* (*P. gingivalis*) and *Tannerella forsythia* (*T. forsythia*) [4].

In addition, peri-implantitis was linked with opportunistic pathogens such as *Pseudomonas aeruginosa* and *Staphylococcus aureus* (*S. aureus*), thus pointing to a rather complex and heterogenous ‘polymicrobal infection’. Yeasts are frequently isolated from the oral cavity [8] and were also identified in the submucosal plaque of patients with peri-implantitis [9,10]. These studies, however, mainly focused on the assessment of *Candida albicans*, and at the time being, a qualitative evaluation of other fungal organisms is lacking. Moreover, the potential role of yeasts and their co-aggregation with either periodontopathogens or other opportunistic bacteria at peri-implantitis sites is unknown.

Therefore, the aim of the present study was to analyze and correlate fungal organisms and bacterial species at peri-implantitis and healthy implant sites as well as teeth with a history of periodontitis using real-time polymerase chain reaction (PCR).

## Methods

### Study population

A total of 29 partially or fully edentulous patients were consecutively recruited from the Department of Oral Surgery, Heinrich Heine University, Düsseldorf, Germany, between April 2013 and July 2014. Nineteen patients (7 men and 13 women; mean age 58.8 ± 12.6 years) suffered from initial to moderate or advanced peri-implantitis, while ten patients (6 men and 4 women; mean age 55.2 ± 11.3 years) revealed clinically healthy implant sites. Prior to participation, each patient was given a detailed description of the procedure and was required to sign informed consent forms. The study was in accordance with the Helsinki Declaration of 2008 and the study protocol was approved by the ethics committee of the Heinrich Heine University.

### Patient selection

For patient selection, the following inclusion criteria were defined: 1) partially or fully edentulous, 2) presence of one screw-type titanium implant either exhibiting healthy (absence of bleeding on probing (BOP), probing depth (PD) <4 mm) or established peri-implantitis (i.e., bleeding on probing with or without suppuration/pus, pocketing, and radiographic bone loss - initial to moderate: <50%/advanced: >50% of the implant length relative to baseline) [11], 3) presence of a sufficiently dimensioned (>2 mm) keratinized mucosa, 4) no implant mobility, 5) no systemic antibiotic medication within the last 3 months, 6) no history of malignancy, radiotherapy, chemotherapy, or immunodeficiency within the last 4 years, 7) proper recall/periodontal maintenance care, 8) non-smoker or light smoking status in smokers (<10 cigarettes per day).

### Plaque samples

After a gentle supramucosal cleaning, submucosal plaque samples and peri-implant sulcus fluid were collected at the deepest aspect of each implant site by means of sterile paper points (i.e., each was left in place for 30 s). The paper point was transferred into 200 μl G2 buffer of the EZ1 DNA Tissue Kit (Qiagen, Hilden, Germany) and stored at −20°C until transportation to the Institute of Medical Microbiology and Hospital Hygiene at the Heinrich Heine University for analysis.

In the peri-implantitis group, one additional subgingival plaque sample was obtained from partially edentulous patients with a history of periodontitis (*n* = 10) and obtained at a tooth exhibiting the highest PD but no signs of acute periodontal disease (i.e., BOP/no suppuration). None of these teeth were located adjacent to the sampled implant sites. The control samples were also prepared for PCR analysis.

### Genomic DNA preparation

At the Institute of Medical Microbiology and Hospital Hygiene, the specimens were re-suspended in the buffer by vortexing. After the addition of 10 μl Proteinase K solution (100 μg/ml Proteinase K), the samples were incubated for 30 min at 56°C. Total genomic DNA was isolated from 200 μl of the Proteinase K-digested samples by semiautomatic DNA preparation on an EZ1 biorobot machine (Qiagen) and the eluted 100 μl DNA samples stored at −20°C until use.

### TaqMan PCR

*In house* TaqMan PCRs for the quantification of *Mycoplasma salivarium* (*M. salivarium*) [12], *Veillonella parvula* (*V. parvula*) [13], *S. aureus* [14], *P. gingivalis* [15], *Parvimonas micra* (*P. micra*) [16], and *T. forsythia* [15] (Table 1) were carried out in a total volume of 25 μl consisting of 1× Eurogentec qPCR MasterMix (Eurogentec, Seraing, Belgium) without ROX (containing buffer, dNTPs (including dUTP), HotGoldStar DNA polymerase, 5 mM MgCl_2_, uracil-N-glycosylase and stabilizers (RT-QP2X-03NR, Eurogentec)), 300 nM each forward and reverse primer, 200 nM labeled probe, and 2.5 μl of template DNA (primer and probes are listed in Tables 1 and 2). Amplicon carrying plasmids were used in concentrations of 10^5^ and 10^2^ copies/μl as quantification standards. Thermal cycling conditions were as follows: 1 cycle at 50°C for 10 min, 1 cycle at 95°C for 10 min followed by 45 cycles at 95°C for 15 s, and 60°C for 1 min. Cycling and fluorescent data collection and analysis were carried out with an iCycler from BioRad (BioRad Laboratories, Munich, Germany) according to the manufacturer’s instructions.

**Table 1:** **Overview of bacterial species, corresponding genes, primers/probes, and DNA sequences**

**Species**	**Gene**	**Primer/probe**	**Sequences (5′-3′)**
*Mycoplasma salivarium*	*rpo*B	msali-F	CCG TCA AAT GAT TTC GAT TGC
		msali-R	GAA CTG CTT GAC GTT GCA TGT T
		msali-S	Hex-ATG ATG CTA ACC GTG CGC TTA TGG GTG-BHQ1
*Veillonella parvula*	16S rDNA	vpar-F	TGC TAA TAC CGC ATA CGA TCT AAC C
		vpar-R	GCT TAT AAA TAG AGG CCA CCT TTC A
		vpar-S	HEX-CTA TCC TCG ATG CCG A-BHQ1
*Staphylococcus aureus*	*nuc*	saur-F	CAA AGC ATC CTA AAA AAG GTG TAG AGA
		saur-R	TTC AAT TTT CTT TGC ATT TTC TAC CA
		saur-S	FAM-TTT TCG TAA ATG CAC TTG CTT CAG GAC CA-BHQ1
*Porphyromonas gingivalis*	Arg-gingipain	pgin-F	CCT ACG TGT ACG GAC AGA GCT ATA
		pgin-R	AGG ATC GCT CAG CGT AGC ATT
		pgin-S	TexRed-TCG CCC GGG AAG AAC TTG TCT TCA-BHQ2
*Parvimonas micra*	16S rDNA	pmic-F	TCG AAC GTG ATT TTT GTG GAA A
		pmic-R	GGT AGG TTG CTC ACG TGT TAC TCA
		pmic-S	FAM-CCC GTT CGC CAC TT-BHQ1
*Tannerella forsythia*	bspA	tfor-F	TCC CAA AGA CGC GGA TAT CA
		tfor-R	ACG GTC GCG ATG TCA TTG T
		tfor-S	FAM-CCG CGA CGT GAA ATG GTA TTC CTC-BHQ1
		tfor-S II	HEX-TCG CGA CGT GAA ATG GTA TTC CTC-BHQ1

**Table 2:** **Overview of fungal organisms, corresponding genes, primers/probes, and DNA sequences**

**Species**	**Gene**	**Primer/probe**	**Sequences (5′-3′)**
*Aspergillus* spp*.* plus	ITS2	aspe-F	CTG TCC GAG CGT CAT TG
*Penicillium* spp*.*		pen1-F	GTC CGA GCG TCA TTT CTG
		pen2-F	TCC GAG CGT CAT TGC TG
		its2-R	TCC TCC GCT TAT TGA TAT GC
*Rhizomucor* spp*.* plus	ITS2	muco-F	GAA CGC AWC TTG CGC TCA
*Mucor* spp*.* plus		rhi1-F	TCA TCC ATT GGG TAC GTC TAG
*Rhizopus* spp*.*		its2-R	TCC TCC GCT TAT TGA TAT GC
*Absidia* spp*.* plus	ITS2	absi-F	ATY TTT GAA CGC ATC TTG CA
*Cunninghamella* spp*.* plus		lic1-F	ATT YAG TTG CTG TCA TGG CC
*Lichtheimia* spp*.*		lic2-F	CAT TCA GTT GCT CTC ATG GTC
		its2-R	TCC TCC GCT TAT TGA TAT GC
*Candida* spp*.*	ITS2	cand-F	CCT GTT TGA GCG TCR TTT
		its2-R	TCC TCC GCT TAT TGA TAT GC

### Real-time PCR

Real-time PCR assays for the detection of fungal DNA (Table 2) were carried out in a total volume of 25 μl consisting of 1× MesaGreen qPCR MasterMix Plus for SYBR Assay (containing Buffer, dNTPs (including dUTP), Meteor Taq DNA polymerase, 4 mM MgCl2, uracil-N-glycosylase, SYBR Green I, stabilizers and passive reference (RT-SY2X-06 + WOU); Eurogentec, Seraing, Belgium), 300 nM each forward and reverse primer and 2.5 μl of template DNA. In multiplex assays with three forward primers, each primer was adjusted to 100 mM. Positive detection was verified by sequencing [17] and BLAST analysis [18]. Thermal cycling conditions were as follows: 10 min at 50°C, 10 min at 95°C followed by 40 cycles of 15 s at 95°C, and 1 min at 60°C. Subsequent melting point analysis followed after 15 s at 95°C and 1 min at 60°C from 65°C to 95°C with an increment of 0.5°C for 15 s and plate read*.*

### Statistical analysis

The statistical analysis was performed using a commercially available software program (SPSS Statistics 22.0, IBM Corp., Ehningen, Germany). Kendall-Tau-b correlation coefficients were calculated to evaluate the dependence between fungal organisms, bacterial species as well as disease severity (i.e., initial to moderate and advanced sites). Results were considered statistically significant at *P* < 0.05.

## Results

According to the given definition, the present analysis was based on a total of *n* = 13 initial to moderate and *n* = 6 advanced peri-implantitis lesions (*n* = 19 patients), 10 healthy implant sites (*n* = 10 patients), as well as 10 teeth with a history of periodontitis (*n* = 10 out of 19 patients suffering from peri-implantitis).

### Fungal and bacterial analysis

The analysis of fungal organisms as well as of *M. salivarium*, *V. parvula*, *S. aureus*, *P. gingivalis*, *P. micra*, and *T. forsythia* at peri-implantitis as well as healthy implant and selected tooth sites is presented in Tables 3, 4, and 5.

**Table 3:** **Bacterial and fungal analysis: peri-implantitis sites (** ***n*** **= 19 patients)**

**Patient**	**Severity**	***M. salivarium***	***V. parvula***	***S. aureus***	***P. gingivalis***	***P. micra***	***T. forsythia***	**Fungal organisms**
1	i-m	-	7.96E + 04	-	-	6.29E + 01	2.14E + 01	*Paelicomyces* spp*.*
2	i-m	3.31E + 02	4.59E + 03	-	-	1.94E + 04	6.60E + 01	-
3	i-m	1.80E + 01	5.25E + 02	-	3.07E + 05	7.67E + 04	9.28E + 04	-
4	i-m	1.02E + 01	3.23E + 03	-	1.16E + 02	1.98E + 02	3.33E + 01	*Candida boidinii, Penicillium* spp*.*
5	i-m	-	2.08E + 03	-	-	1.11E + 01	1.51E + 01	-
6	i-m	1.23E + 02	2.29E + 03	-	4.40E + 04	1.45E + 03	1.33E + 04	-
7	i-m	1.17E + 03	9.02E + 02	-	6.37E + 03	3.71E + 03	4.28E + 01	-
8	i-m	-	3.13E + 03	-	-	-	3.87E + 00	*Candida albicans*
9	i-m	-	1.13E + 02	-	7.67E + 01	4.26E + 03	5.20E + 03	-
10	i-m	1.46E + 04	5.58E + 04	-	1.24E + 01	1.29E + 03	5.33E + 04	-
11	i-m	-	8.22E + 03	-	-	-	1.06E + 02	-
12	i-m	1.48E-01	4.98E + 03	37.43	2.31E + 02	3.10E + 01	5.33E + 01	*Cladosporium cladosporioides*
13	i-m	8.46E + 02	1.76E + 02	-	-	1.33E + 04	2.99E + 03	*-*
14	a	1.66E + 03	3.90E + 04	-	1.28E + 05	6.75E + 03	1.02E + 04	*Rhodotorula laryngis*, *Candida alb.*
15	a	1.91E + 02	1.13E + 03	-	1.51E + 01	6.33E + 03	1.56E + 01	
16	a	1.29E + 05	1.27E + 04	-	-	3.85E + 05	1.29E + 05	-
17	a	6.82E + 01	5.43E + 01	-	-	3.00E + 04	1.17E + 04	-
18	a	1.95E + 04	1.35E + 05	-	1.46E + 04	2.25E + 03	4.44E + 03	-
19	a	-	7.43E + 03	-	3.08E + 01	1.26E + 02	1.33E + 02	-

i-m, initial to moderate; a, advanced.

**Table 4:** **Bacterial and fungal analysis: healthy implant sites (** ***n*** **= 10 patients)**

**Patient**	***M. salivarium***	***V. parvula***	***S. aureus***	***P. gingivalis***	***P. micra***	***T. forsythia***	**Fungal organisms**
1	-	2.26E + 03	-	1.64E + 01	8.02E + 00	7.58E + 01	-
2	1.46E + 04	3.94E + 04	36.45	-	4.00E + 04	1.14E + 04	*Candida dubliniensis*
3	-	3.16E + 04	37.49	1.62E + 01	8.88E + 01	4.90E + 01	-
4	-	3.42E + 04	-	-	1.62E + 04	7.78E + 01	-
5	-	1.78E + 04	-	-	1.81E + 02	3.66E + 03	-
6	-	2.74E + 03	-	-	1.38E + 01	1.40E + 02	*Cladosporium cladosporioides*
7	-	2.50E + 02	36.90	-	1.03E + 03	6.19E + 01	-
8	9.34E + 01	1.37E + 03	36.61	8.52E + 02	1.15E + 03	2.84E + 01	-
9	2.04E + 01	1.29E + 03	-	-	8.06E + 01	8.79E + 02	*Cladosporium cladosporioides*
10	-	-	-	-	4.48E + 02	8.69E + 02	*Cladosporium cladosporioides*

**Table 5:** **Bacterial and fungal analysis: tooth sites (** ***n*** **= 10 patients)**

**Patient**	***M. salivarium***	***V. parvula***	***S. aureus***	***P. gingivalis***	***P. micra***	***T. forsythia***	**Fungal organisms**
1	7.05E + 01	7.00E + 01	-	-	-	4.67E + 01	-
5	-	1.18E + 04	-	6.30E + 00	5.02E + 02	1.51E + 01	-
6	4.08E + 03	6.71E + 02	-	-	5.23E + 03	4.89E + 04	*Fusarium solani*
7	4.55E + 02	7.06E + 03	-	1.43E + 05	1.47E + 04	6.36E + 04	-
10	-	1.79E + 02	-	1.76E + 01	5.49E + 00	2.40E + 00	-
12	1.79E + 03	1.92E + 03	-	-	1.43E + 04	3.59E + 03	*Candida albicans*
13	-	1.26E + 02	-	-	-	1.21E + 02	-
16	-	3.19E + 03	-	1.07E + 04	3.44E + 03	3.01E + 04	-
17	2.69E + 01	2.68E + 03	28.25	6.82E + 01	2.08E + 02	4.83E + 01	-
19	1.03E + 01	1.33E + 02	-	-	9.81E + 01	4.08E + 01	-

#### Peri-implantitis sites

Fungal organisms were identified in 31.6% (six sites) of the patients and equally distributed between initial to moderate (three sites) and advanced (two sites) peri-implantitis sites. The respective plaque samples were dominated (*n* = 3) by *Candida* spp*.* (i.e., *C. albicans* and *Candida boidinii*) and at two sites co-colonized with *Penicillium* spp*.* and *Rhodotorula laryngis. Paelicomyces* spp*.* (67% homologous), *Saccharomycetes* (76% homologous), and *Cladosporium cladosporioides* were identified at three sites (Table 3). The Kendall-Tau-b coefficients failed to reveal any significant correlations between the presence of fungal organisms and the proportions of *M. salivarium* (−0.26), *V. parvula* (0.26), *S. aureus* (0.34), and *P. gingivalis* (0.09) as well as disease severity (0.26) (*P* > 0.05, respectively). However, a significant correlation was noted with respect to the proportions of *P. micra* (−0.42) and *T. forsythia* (−0.44) (*P* < 0.05, respectively).

#### Healthy implant sites

In the selected partially edentulous patients, fungal organisms were identified at four implant sites (*Candida dubliniensis* and *C. cladosporioides*), corresponding to a frequency of 40.0% (Table 4). The Kendall-Tau-b coefficients failed to reveal any significant correlations between the presence of fungal organisms and the proportions of *M. salivarium* (0.38), *V. parvula* (−0.24), *S. aureus* (−0.33), and *P. gingivalis* (−0.51) (*P* > 0.05, respectively). However, a significant correlation was noted with respect to the proportions of *P. micra* (0.65) and *T. forsythia* (0.65) (*P* < 0.05, respectively).

#### Selected tooth sites

In the selected partially edentulous patients, fungal organisms were identified at two tooth sites (*C. albicans* and *Fusarium solani*), corresponding to a frequency of 20.0% (Table 5).

The Kendall-Tau-b coefficients failed to reveal any significant correlations between the presence of fungal organisms and the proportions of *M. salivarium* (0.25), *V. parvula* (0.34), *P. gingivalis* (0.60), *P. micra* (0.32), *T. forsythia* (0.12), and *S. aureus* (0.66) (*P* > 0.05, respectively).

## Discussion

The present study aimed at analyzing and correlating fungal organisms with several periodontopathogenic and opportunistic bacterial species at peri-implantitis sites using real-time PCR. These outcomes were compared with those noted at healthy implant sites as well as teeth with a history of periodontitis.

Basically, the present analysis has pointed to a high prevalence of fungal organisms in submucosal plaque samples obtained at both peri-implantitis (31.6%) and healthy (40%) implant sites. Peri-implantitis sites were dominated by *Candida* spp*.* (i.e., *C. albicans* and *C. boidinii*) and occasionally co-colonized with *Penicillium* spp*.* and *R. laryngis*, while at three additional sites, *Paelicomyces* spp*.*, *Saccharomycetes*, and *C. cladosporioides* were identified. Healthy implant sites were mainly associated with *C. dubliniensis* and *C. cladosporioides*.

In this context, it must be emphasized that this is the first report on *Penicillium spp.*, *R. laryngis*, *Paelicomyces* spp*.*, *Saccharomycetes*, and *C. cladosporioides* at implant sites, and therefore, any comparison with previous findings is not feasible. However, the high proportions of *Candida* spp*.* noted in the present analysis corroborate previous data also pointing to a frequency of 55% at peri-implantitis sites [9], while a most recent study merely identified *C. albicans* in 3% of the patients investigated [10].

In contrast to the present data, Leonhardt et al. failed to identify *Candida* spp*.* in a total of 51 patients with clinically healthy mucosal conditions [9]. In this context, however, it is also important to emphasize that the presence of *C. albicans* per se does not necessarily cause symptomatic oral mucosal lesions (i.e., stomatitis) [19]. Host susceptibility to these infections is commonly triggered either by local or systemic (e.g., HIV infection, antibiotic medication) factors [20,21]. Furthermore, *Candida* spp*.* possess a high potential to colonize and invade gingival tissues [22] and co-aggregate with other oral microorganisms such as *Pg* [23,24]. The present microbiological analysis also has identified high proportions of periodontopathogenic bacteria associated with peri-implant diseases, thus corroborating previous analyses [4,7,9,25,26]. However, at both peri-implantitis and healthy implant sites, fungal organisms were only correlated with *P. micra* and *T. forsythia*. Unfortunately, the PCR analysis employed did not allow for a quantification of yeasts, and therefore, further studies are needed to determine relative differences in the composition of these specific organisms at healthy and diseased implant sites.

Furthermore, the present analysis failed to identify any significant correlation of either fungal organisms or disease severity with opportunistic bacteria, such as *M. salivarium*, *V. parvula*, and *S. aureus*. At tooth sites, *M. salivarium* was mainly isolated from the sulcus area and associated with gingivitis lesions [27]. Interestingly, *S. aureus* has only been identified at one single peri-implantitis - but several healthy implant sites, which is contradictory to the higher prevalence at peri-implantitis sites noted in larger cohorts [4,7,9]. When further analyzing the present data, it was also noted that at several sites, the frequency of selected periodontopathogenic bacteria was below the detection thresholds, irrespective of disease severity. These findings clearly corroborate previous data indicating that these periodontopathogenic bacteria may not necessarily be related to peri-implantitis [6].

Fungal organisms have also been isolated from periodontal pockets in chronic periodontitis patients. The reported prevalence of yeast-positive samples varied between 15.6% and 17.5% [28,29]. These untreated periodontal pockets were also dominated by *C. albicans*. Other species, such as *Candida parapsilosis*, *C. dubliniensis*, *Candida tropicalis*, and *Rhodotorula* spp*.*, were rarely observed [30]. Even though the prevalence of *C. albicans* tended to be higher in chronic periodontitis (30%) when compared with healthy patients (15%), this difference did not reach statistical significance [30]. The present frequency of fungal organisms in subgingival plaque samples obtained from teeth with a history of periodontitis basically corroborates the above reported data but was markedly lower when compared with all implant sites investigated. However, the frequency distribution of periodontopathogenic and opportunistic bacteria did not seem to differ between tooth and implant sites, which is basically in line with a recent analysis assessing *Aggregatibacter actinomycetemcomitans*, *Pg*, *Prevotella intermedia*, *Tf*, *Treponema denticola*, *S. aureus*, enteric bacteria, and *P. aeruginosa* [10]. In this context, it must be emphasized that the present analysis just focused on the most relevant periodontopathogens associated with peri-implantitis [4] as well as a few opportunistic bacteria [4,7,9,27] that were linked to the oral cavity. Therefore, future analyses should consider a broader spectrum of potential pathogens.

## Conclusions

Within the limitations of the present analysis, it was concluded that *Candida* spp*.* and other fungal organisms were frequently identified at peri-implantitis as well as healthy implant sites and co-colonized with *P. micra* and *T. forsythia*.

## Abbreviations

BOP: Bleeding on probing
*M. salivarium*: *Mycoplasma salivarium*
*P. gingivalis*: *Porphyromonas gingivalis*
*P. micra*: *Parvimonas micra*
PD: Probing pocket depth
*S. aureus*: *Staphylococcus aureus*
*T. forsythia*: *Tannerella forsythia*
*V. parvula*: *Veillonella parvula*
